# Impact of drought on soil microbial biomass and extracellular enzyme activity

**DOI:** 10.3389/fpls.2023.1221288

**Published:** 2023-08-25

**Authors:** Qing Qu, Zhen Wang, Quan Gan, Rentao Liu, Hongwei Xu

**Affiliations:** ^1^ Breeding Base for State Key Laboratory of Land Degradation and Ecological Restoration in Northwestern China, Key Laboratory of Restoration and Reconstruction of Degraded Ecosystems in Northwestern China of Ministry of Education, Ningxia University, Yinchuan, China; ^2^ Institute of Soil and Water Conservation, Chinese Academy of Sciences and Ministry of Water Resources, Yangling, China; ^3^ College of Forestry, Sichuan Agricultural University, Chengdu, China

**Keywords:** biogeochemical cycles, climate change, ecosystem function, ecosystem structure, soil microbial activity, soil microbial community

## Abstract

**Introduction:**

With the continuous changes in climate patterns due to global warming, drought has become an important limiting factor in the development of terrestrial ecosystems. However, a comprehensive understanding of the impact of drought on soil microbial activity at a global scale is lacking.

**Methods:**

In this study, we aimed to examine the effects of drought on soil microbial biomass (carbon [MBC], nitrogen [MBN], and phosphorus [MBP]) and enzyme activity (β-1, 4-glucosidase [BG]; β-D-cellobiosidase [CBH]; β-1, 4-N-acetylglucosaminidase [NAG]; L-leucine aminopeptidase [LAP]; and acid phosphatase [AP]). Additionally, we conducted a meta-analysis to determine the degree to which these effects are regulated by vegetation type, drought intensity, drought duration, and mean annual temperature (MAT).

**Result and discussion:**

Our results showed that drought significantly decreased the MBC, MBN, and MBP and the activity levels of BG and AP by 22.7%, 21.2%, 21.6%, 26.8%, and 16.1%, respectively. In terms of vegetation type, drought mainly affected the MBC and MBN in croplands and grasslands. Furthermore, the response ratio of BG, CBH, NAG, and LAP were negatively correlated with drought intensity, whereas MBN and MBP and the activity levels of BG and CBH were negatively correlated with drought duration. Additionally, the response ratio of BG and NAG were negatively correlated with MAT. In conclusion, drought significantly reduced soil microbial biomass and enzyme activity on a global scale. Our results highlight the strong impact of drought on soil microbial biomass and carbon- and phosphorus-acquiring enzyme activity.

## 1 Introduction

With the intensification of climate warming, global precipitation patterns have changed considerably, affecting the structure, function, and biodiversity of terrestrial ecosystems (van der Molen et al., 2011; Lozano et al., 2021). Data indicate that the occurrence of future extreme weather events, such as rainfall or drought, will likely exhibit a trend of long duration and wide impact (Stott, 2016). In particular, drought will reduce soil moisture, which directly affects plant growth and photosynthesis, thereby affecting the versatility of soil ecosystems (Barnes et al., 2018; Li et al., 2022; Wan et al., 2022).

As the most active component of soil organic matter, the microbial biomass (carbon [MBC], nitrogen [MBN], and phosphorus [MBP]) is very sensitive to changes in the soil environment and can accurately reflect changes in soil carbon and nitrogen content (Wardle, 1998; Bastos et al., 2023). As an important component of soil biological activity, enzymes determine the intensity and direction of various biochemical processes in soil and are an indicator of soil fertility and vitality (Wang et al., 2023a). Soil microbial biomass content and enzyme activity exhibit a more rapid response to changes in soil moisture than to changes in other soil properties. For example, a decrease in soil water availability directly or indirectly affects the reproduction and activity of microorganisms (Ren et al., 2018; Chen et al., 2023). Therefore, exploring the effects of drought on microbial activity will contribute to our understanding of the structure and function of terrestrial ecosystems under various global precipitation patterns.

**Figure 1:**
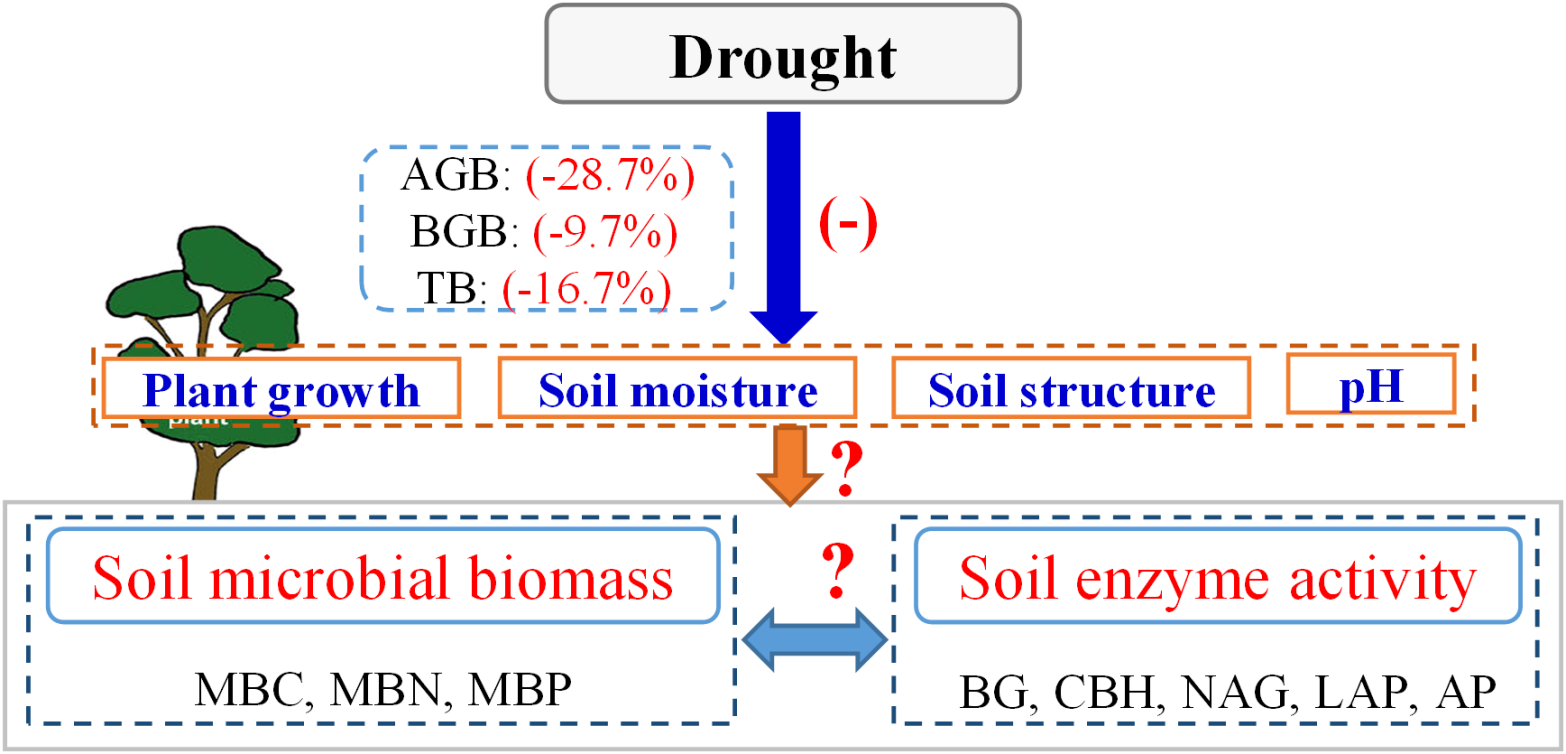
Conceptual framework showing impacts of drought on plant growth, soil microbial biomass, and enzyme activity. “+” and “-” indicate positive and negative effects, respectively. “?”, unresolved by the previous study. Data on the effects of drought on plant biomass comes from the results of the previous meta-analysis (Wang et al., 2021b). AGB, above-ground biomass. BGB, below-ground biomass. TB, total biomass. MBC, soil microbial biomass carbon. MBN, microbial biomass nitrogen. MBP, microbial biomass phosphorus. BG, β-1, 4-glucosidase. CBH, β-D-cellobiosidase. NAG, β-1, 4-N-acetylglucosaminidase. LAP, L-leucine aminopeptidase.

Previous studies have shown that prolonged drought limits vegetation growth and alters microbial community structure (Mishra et al., 2021; Peszek et al., 2021; Wang et al., 2023b). For example, Wang et al. (2021b) demonstrated that drought reduced plant primary productivity and biomass by 12.6% and 16.7%, respectively (**Figure 1**). A reduction in plant biomass directly affects the sources of energy for microorganisms (Song et al., 2019; Ge et al., 2022; Malik and Bouskill, 2022). Additionally, drought affects microbial activity by increasing osmotic stress and resource competition (Canarini et al., 2021; Xie et al., 2021; He et al., 2023). A decrease in soil water availability directly leads to the dehydration of some microorganisms, thereby reducing overall microbial activity, and even leading to the death and decomposition of some microorganisms (Zhang et al., 2023). In addition, lack of soil moisture affects the physiological characteristics of microorganisms and reduces their ability to acquire and utilise pairs (Sistla and Schimel, 2012), thus limiting their biological activity owing to a lack of energy. However, Sanaullah et al. (2011) found that drought did not substantially reduce the soil microbial biomass; however, this result may be related to the strong drought resistance of crops or local climatic conditions in this study. Therefore, uncertainties remain regarding the impacts of drought on microbial activity (**Figure 1**). A meta-analysis is urgently needed to determine these effects and to reveal the response of terrestrial biogeochemical cycles to changes in global precipitation patterns.

Drought intensity and duration are important factors affecting microbial activity (Sun et al., 2020). Moderate drought inhibits plant growth; however, an excessive reduction in soil water availability may lead to plant death (Hoover et al., 2014; Akram et al., 2020; Zang et al., 2020). A recent meta-analysis confirmed this conclusion (Wang et al., 2021b). Substantial reductions in plant biomass limit the availability of food sources for microorganisms (Brown et al., 2021). Furthermore, excessive drought damages soil properties, such as structure, porosity, and pH (Chen et al., 2020; Lozano et al., 2021; Peng et al., 2023), thereby creating environmental conditions that are unfavourable for the growth of microorganisms. Consequently, microbial metabolism, activity, and enzyme production are reduced (Manzoni et al., 2014; Fitzpatrick et al., 2017).

Furthermore, excessive drought damages soil properties, such as structure, porosity, and pH (Chen et al., 2020; Lozano et al., 2021; Peng et al., 2023), thereby creating environmental conditions that are unfavourable for the growth of microorganisms. Consequently, microbial metabolism, activity, and enzyme production are reduced (Manzoni et al., 2014; Fitzpatrick et al., 2017). For example, compared with those in forests, plants in croplands and grasslands typically have lower biomass and shallower root systems (Jian et al., 2015; Cheng et al., 2016); therefore, after drought, there are large differences in the effects on plant growth for each vegetation type, resulting in different impacts on soil microorganisms. However, owing to the complexity and heterogeneity of ecosystems (Hu et al., 2021; Wang et al., 2021a), our understanding of how drought affects soil microbial activity under different vegetation types is still incomplete. In addition, microbial activity is also regulated by climatic factors such as mean annual temperature (MAT) (Malik et al., 2020a). Moderate temperature increases can promote plant growth and accelerate soil nutrient turnover (Akram et al., 2022; Joly et al., 2023), but excessively high temperatures may exacerbate soil moisture loss (Qu et al., 2023), resulting in strong inhibition of microbial activity. Overall, research on the global-scale impacts of drought on microbial activity, especially in terms of drought intensity, drought duration, vegetation type, and MAT is lacking (**Figure 1**). This gap in the existing literature has limited our understanding of the impacts of increasing drought on ecosystem structure, function, and biodiversity under global climate change.

Based on meta-analysis and published data, this study investigated the effects of drought on soil microbial activity. The specific aims of this study were to identify: (1) how drought affects soil microbial biomass and enzyme activity and (2) whether this effect is regulated by vegetation type (cropland, grassland, shrub, and forest), geographical location/continents (Asia, America, Europe, Oceania, and Africa), drought intensity, drought duration, and MAT. Our hypotheses were as follows: (1) Drought inhibits soil microbial activity due to adverse soil environmental conditions (e.g., reduced availability of energy sources and changes in physical structure) (Manzoni et al., 2014; Fitzpatrick et al., 2017). (2) The effect of drought on microbial activity is higher in croplands and grasslands than in forests owing to different plant growth characteristics (Clark et al., 2009). (3) Drought intensity and duration aggravate its negative effects on microbial activity because severe and prolonged droughts can cause plants to wither and die, thereby reducing plant biomass (Smith et al., 2009; Brown et al., 2021). The results of this study contribute to an improved understanding of the impact of drought on the structure, function, and biodiversity of terrestrial ecosystems.

## 2 Materials and methods

### 2.1 Data collection

We utilized the Web of Science, Google Scholar, and the China National Knowledge Infrastructure databases to search all relevant literature published prior to 2023. The search terms are listed in **Supplementary Table 1**. After a preliminary screening of titles and abstracts, the literature was evaluated again based on the following criteria: (1) A treatment group (drought) and control group (normal water supply) must be included. (2) The vegetation types, drought intensity, and drought duration must be identified. (3) At least one research index must be included (**Supplementary Table 2**). (4) The research should not include the interaction of multiple factors, such as nitrogen addition, warming, or carbon dioxide doubling. (5) If data for multiple soil layers were reported in the study, only the manifested soil index data were obtained. (6) The research must clarify the mean, sample size, and standard deviation (SD) of all variables. If no standard deviation was reported, it was calculated using the standard error (SE) as follows: $\text{SD}=\text{SE}\sqrt{\text{N}}$ (Fu et al., 2015). The screening steps are shown in **Supplementary Figure 1**. In addition, we collected data on longitude, latitude, MAT, mean annual precipitation (MAP), drought intensity, and drought duration. MAT, mean annual precipitation (MAP) are obtained directly from the article or from the WorldClim database (http://www.worldclim.org/).

### 2.2 Meta-analysis

The response ratio (RR) was used to measure the influence of drought on related variables (Hedges et al., 1999), and was calculated using the following formula:


(1) $$\text{RR}=\text{ln}\left(\frac{\bar{X_{t}}}{\bar{X_{c}}}\right)=ln\bar{X_{t}}-ln\bar{X_{c}}$$

(2) $$v=\frac{S_{t}^{2}}{n_{t}X_{t}^{2}}+\frac{S_{c}^{2}}{n_{c}X_{c}^{2}}$$

where, $\bar{X_{t}}$ and $\bar{X_{c}}$ are the mean values of the variables in the experimental and control groups, respectively; nt and nc are the sample sizes of the variables in the experimental and control groups, respectively; and St and Sc are the SD of the variables in the experimental and control groups, respectively.

The weighted response ratio ( $RR_{++}$ ), 95% bootstrap confidence interval (CI), standard error $S\left(RR_{++}\right)$ , and weighting factor (w) were calculated using the random-effects model. If the 95% bootstrap CI was located to the left of the zero-carving line, it indicated that, compared with the control group, the treatment group had a negative effect on related research indicators; otherwise, it had a positive effect. When zero was included, drought had no significant influence on the corresponding variables. These values were calculated using the following equations:


(3) $$RR_{++}=\frac{\sum_{i=1}^{m}\sum_{j=1}^{ki}W_{ij}RR_{ij}}{\sum_{i=1}^{m}\sum_{j=1}^{ki}W_{ij}}$$

(4) $$95\text{\%CI}=RR_{++}\pm 1.96S\left(RR_{++}\right)$$

(5) $$S\left(RR_{++}\right)=\sqrt{\frac{1}{\sum_{i=1}^{m}\sum_{j=1}^{ki}W_{ij}}}$$

(6) $$w_{ij}=\frac{1}{\vartheta_{i}+\sigma^{2}}$$

where, $\vartheta_{i}$ and $\sigma^{2}$ are the variance of the data in the i-th study and the random variable that exists between the studies, respectively.

To describe the RR of each variable more intuitively and clearly, we converted the value to a percentage using the following formula:


(7) $$\text{Effect size }\left(\%\right)=\left[exp\left(RR_{++}\right)-1\right]\times 100\%$$

In this study, a linear mixed model was used to analyse whether the RRs of soil microbial biomass and enzyme activity were affected by vegetation type (cropland, grassland, shrub, and forest) and continents (Asia, America, Europe, Oceania, and Africa). “Study” was designated as the random effects component (Bates et al., 2015; Hao et al., 2022). The influence of the grouping variables on microbial activity was calculated using the random-effects model, which indicated heterogeneity in the group cumulative effect sizes (Q_M_) (Gao et al., 2021; Xu et al., 2022a). Regression analysis was selected to study the relationships of the RRs of soil microbial biomass and enzyme activity with drought intensity, drought duration, and MAT. A funnel plot was used to assess potential publication bias (**Supplementary Figure 2**). The above processes were performed using the R v.4.0.2 metafor package. Both integrated and regression analysis diagrams were completed using Origin 9.0.

## 3 Results

### 3.1 Overview of the dataset

**Figure 2:**
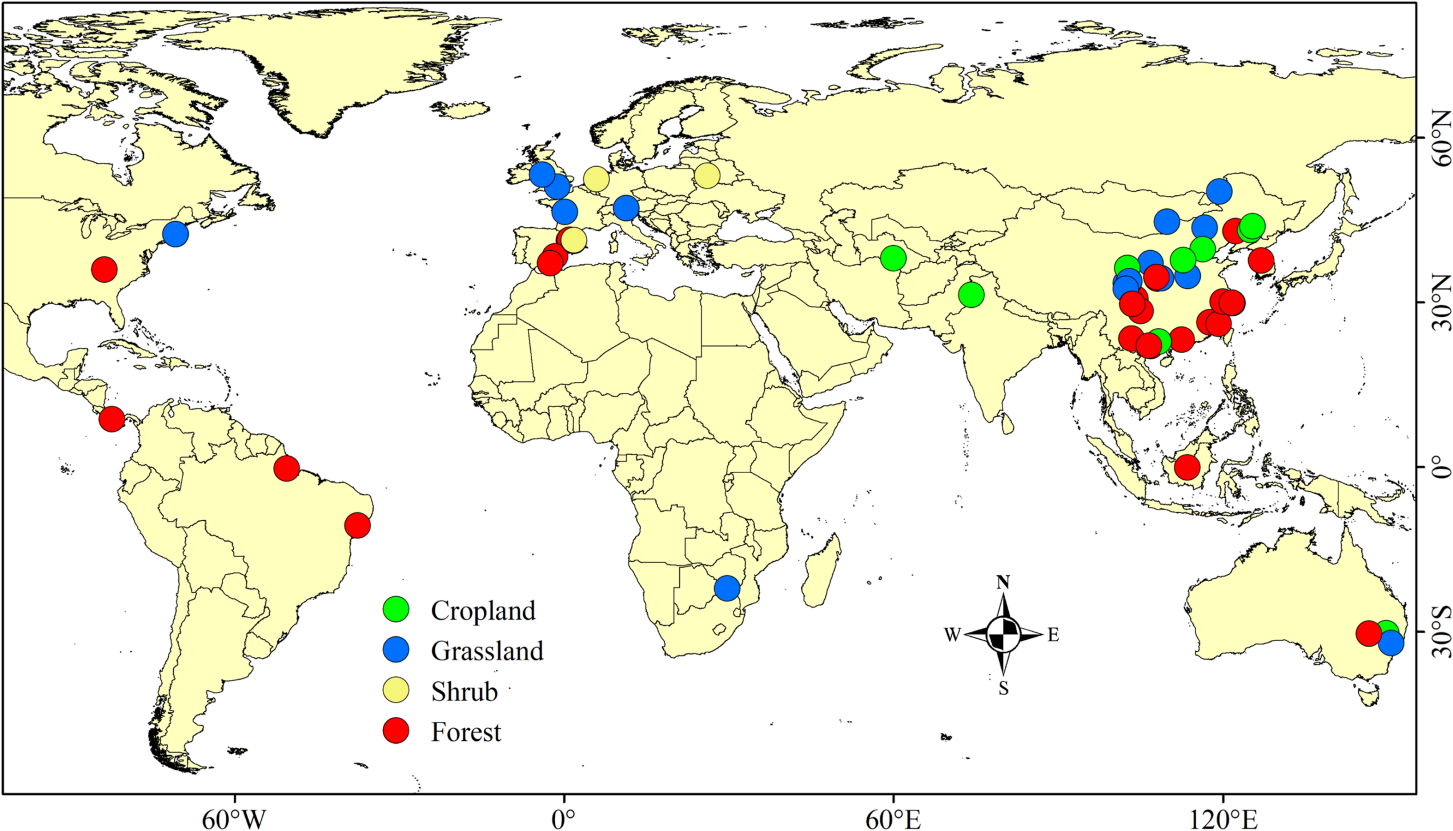
Global distribution of study sites used in this meta-analysis.

In total, 60 studies encompassing 250 data points and 12 variables were included in this study (Appendix Dataset 1, **Supplementary Table 2**). The sample sizes of cropland, grassland, shrub, and forest were 41, 92, 11, and 106, respectively. The distribution of the sample points is shown in **Figure 2**.

### 3.2 Overall effects

**Figure 3:**
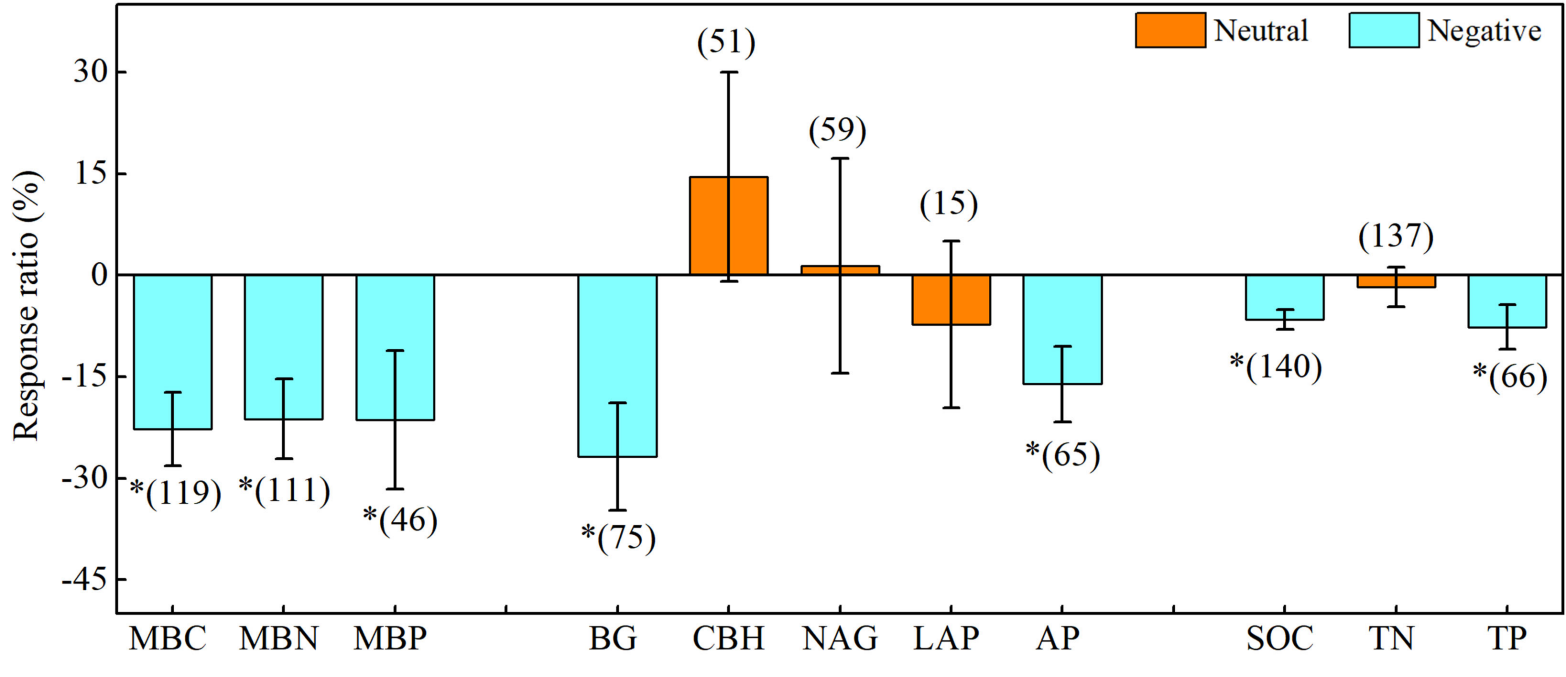
Effects of drought on soil microbial biomass, enzyme activity, and soil chemistry properties. Error bars denote the 95% confidence interval (CI). The number in parentheses indicates the sample size. * indicates that the impact of drought is considered as significant (*p*< 0.05). MBC, soil microbial biomass carbon. MBN, microbial biomass nitrogen. MBP, microbial biomass phosphorus. BG, β-1, 4-glucosidase. CBH, β-D-cellobiosidase. NAG, β-1, 4-N-acetylglucosaminidase. LAP, L-leucine aminopeptidase. AP, acid phosphatase. SOC, soil organic carbon. TN, total nitrogen. TP, total phosphorus.

Drought significantly affected soil microbial biomass and enzyme activity, and the effects varied among different variables (**Figure 3**). Briefly, drought significantly decreased soil microbial biomass carbon (MBC), microbial biomass nitrogen (MBN), and microbial biomass phosphorus (MBP) by 22.7%, 21.2%, and 21.6%, respectively. Meanwhile, drought considerably decreased the activities of β-1, 4-glucosidase (BG) and acid phosphatase (AP) by 26.8% and 16.1%, respectively, but had a lesser effect on β-D-cellobiosidase (CBH), β-1, 4-N-acetylglucosaminidase (NAG), and L-leucine aminopeptidase (LAP). Additionally, drought markedly decreased the soil organic carbon (SOC) and total phosphorus (TP) by 6.5% and 7.6%, respectively. Overall, drought had a stronger inhibitory effect on soil microbial biomass and soil carbon- and phosphorus-acquiring enzyme activity and a weaker effect on soil nitrogen-acquiring enzyme activity.

### 3.3 Response of soil microbial biomass and enzyme activity to vegetation types and continents

**Figure 4:**
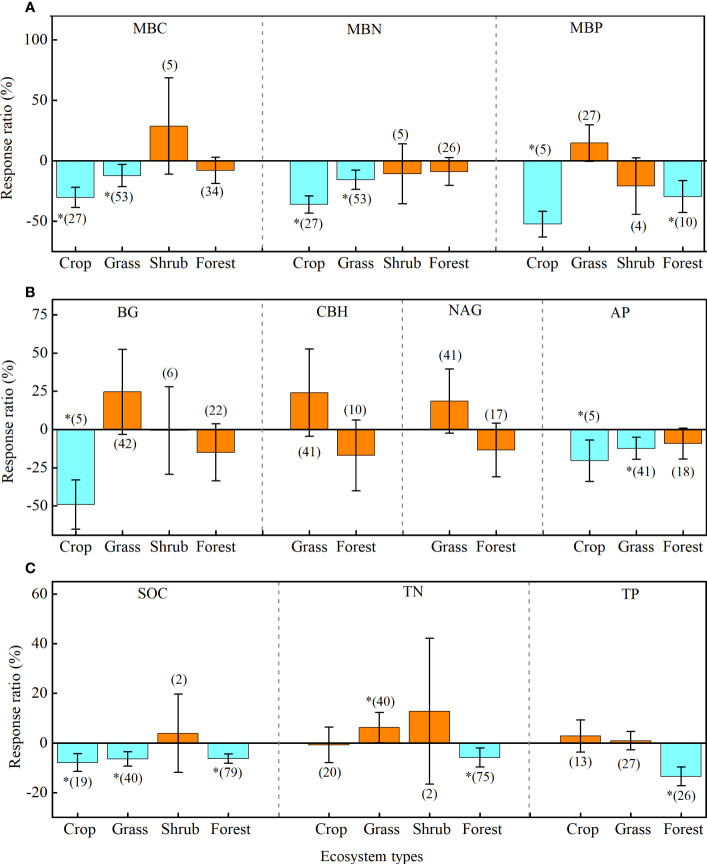
Effects of drought on soil microbial biomass **(A)**, enzyme activity **(B)**, and soil chemistry properties **(C)** of four ecosystem types: cropland (Crop), grassland (Grass), shrub, and forest. Error bars denote the 95% confidence interval (CI). The number in parentheses indicates the sample size. * indicates that the impact of drought is considered as significant (*p*< 0.05). MBC, soil microbial biomass carbon. MBN, microbial biomass nitrogen. MBP, microbial biomass phosphorus. BG, β-1, 4-glucosidase. CBH, β-D-cellobiosidase. NAG, β-1, 4-N-acetylglucosaminidase. LAP, L-leucine aminopeptidase. AP, acid phosphatase. SOC, soil organic carbon. TN, total nitrogen. TP, total phosphorus.

The effects of drought on soil microbial biomass and enzyme activity are regulated by vegetation types (**Figure 4**; **Supplementary Table 3**). Briefly, drought negatively affected MBC and MBN in croplands (MBC: -30.2%; MBN: -36.1%) and grasslands (MBC: -12.1%; MBN: -15.6%), but it had less effect on these factors in shrubs and forests (**Figure 4A**). Drought negatively affected BG in croplands (-49.1%) and AP in croplands (-20.5%) and grasslands (-12.6%), whereas the effect on CBH and NAG were all neutral in grasslands and forests (**Figure 4B**). In addition, drought negatively affected SOC in croplands (-7.9%), grasslands (-6.3%), and forests (-6.4%) (**Figure 4C**).

**Figure 5:**
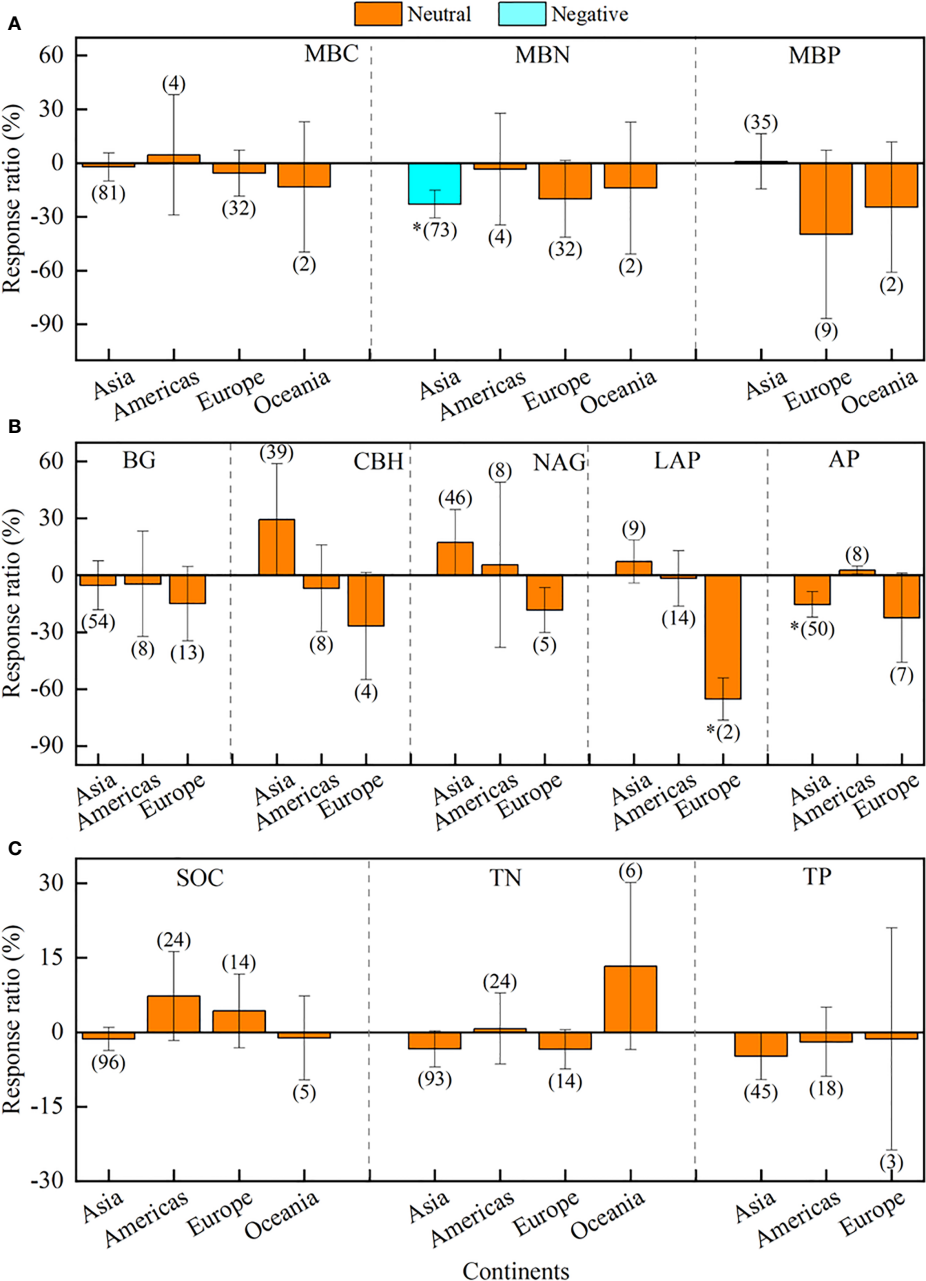
Effects of different continents of Asia, America, Europe, Oceania, and Africa on soil microbial biomass **(A)**, enzyme activity **(B)**, and soil chemistry properties **(C)**. Error bars denote the 95% confidence interval (CI). The number in parentheses indicates the sample size. * indicates that the impact of drought is considered as significant (*p*< 0.05). MBC, soil microbial biomass carbon. MBN, microbial biomass nitrogen. MBP, microbial biomass phosphorus. BG, β-1, 4-glucosidase. CBH, β-D-cellobiosidase. NAG, β-1, 4-N-acetylglucosaminidase. LAP, L-leucine aminopeptidase. AP, acid phosphatase. SOC, soil organic carbon. TN, total nitrogen. TP, total phosphorus.

In contrast, continents played a smaller role in regulating the effects of drought on soil microbial biomass and enzyme activity (**Figure 5**; **Supplementary Table 4**). Briefly, drought only negatively affected MBN in Asia (-22.9%), LAP in Europe (-65.2%), and AP in Asia (-15.4%), whereas the effects on other indices were neutral in all continents (**Figures 5A–C**).

### 3.4 Response of soil microbial biomass and enzyme activity to drought intensity and duration

**Figure 6:**
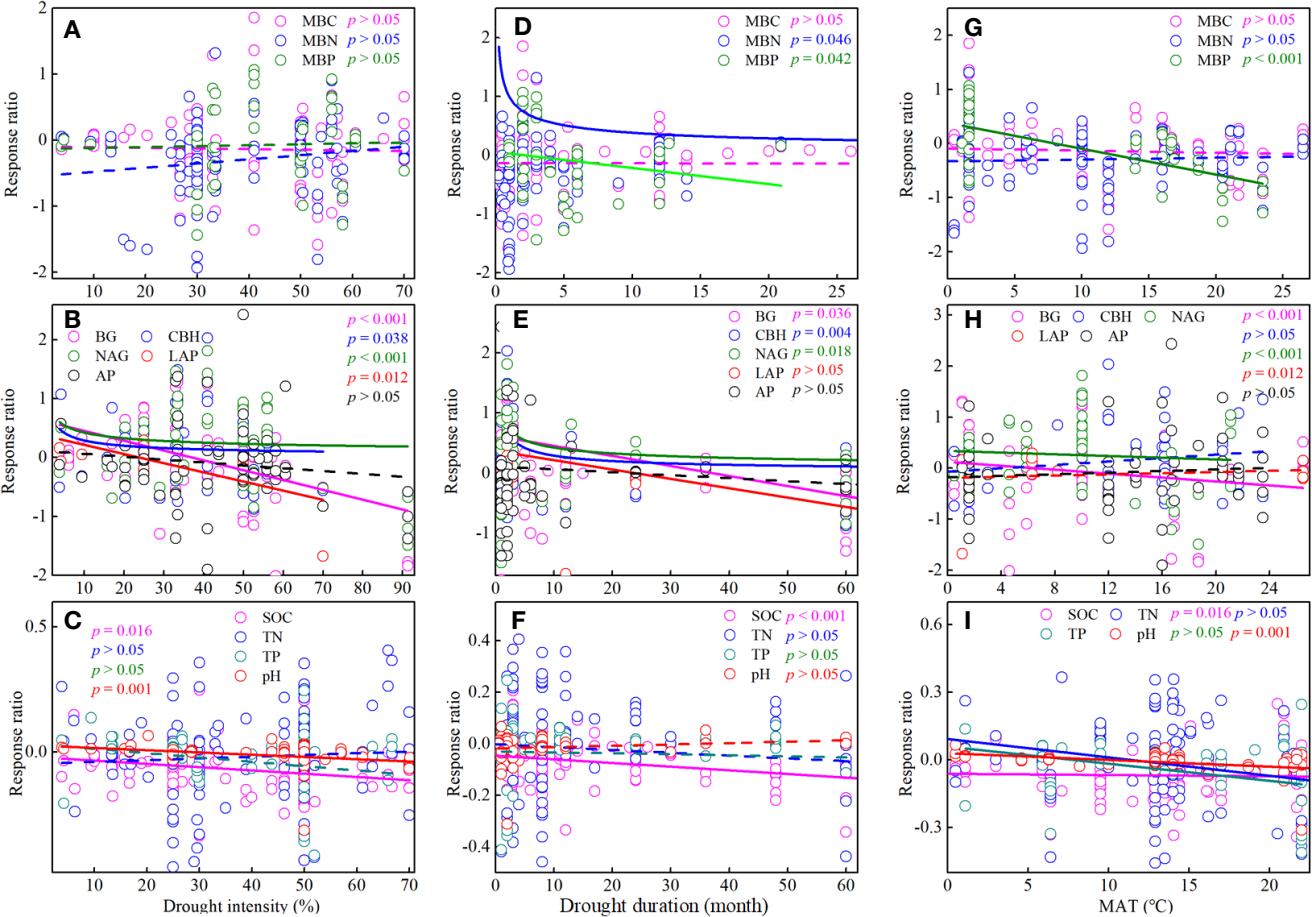
Effects of drought intensity **(A-C)**, drought duration **(D-F)**, and annual average temperature (MAT) **(G-I)** on soil microbial biomass, enzyme activity, and soil chemistry properties. MBC, soil microbial biomass carbon. MBN, microbial biomass nitrogen. MBP, microbial biomass phosphorus. BG, β-1, 4-glucosidase. CBH, β-D-cellobiosidase. NAG, β-1, 4-N-acetylglucosaminidase. LAP, L-leucine aminopeptidase. AP, acid phosphatase. SOC, soil organic carbon. TN, total nitrogen. TP, total phosphorus.

Drought intensity had different effects on soil microbial biomass and enzyme activity (**Figure 6**). Briefly, the response ratio of BG, CBH, NAG, and LAP decreased significantly (p< 0.05) with increasing drought intensity, whereas the response ratio of soil microbial biomass did not show significant (p > 0.05) changes with increasing drought intensity (**Figures 6A, B**). Meanwhile, drought intensity did not significantly affect the response ratio of total nitrogen (TN) and TP (p > 0.05), but it was significantly negatively correlated with the response ratio of SOC and pH (**Figure 6C**).

Drought duration also showed different effects on soil microbial biomass and enzyme activity (**Figure 6**). Briefly, the response ratio of MBN, MBP, BG, CBH, NAG, and LAP decreased significantly (p< 0.05) with increasing drought duration, whereas the response ratio of MBC did not show significant (p > 0.05) changes with increasing drought duration (**Figures 6D, E**). Meanwhile, the response ratio of SOC also decreased significantly with increasing drought duration (**Figure 6F**).

### 3.5 Response of soil microbial biomass and enzyme activity to MAT

The responses of soil microbial biomass and enzyme activity to drought were influenced by MAT (**Figure 6**). Briefly, the response ratio of MBP, BG, and NAG decreased significantly (p< 0.05) with MAT, whereas the response ratio of MBC, MBN, CBH, LAP, and AP did not show significant (p > 0.05) changes with MAT (**Figures 6G, H**). Meanwhile, the response ratio of SOC, TN, TP, and pH also decreased significantly with MAT (**Figure 6I**).

## 4 Discussion

### 4.1 Effect of drought on soil microbial biomass and enzyme activity

**Figure 7:**
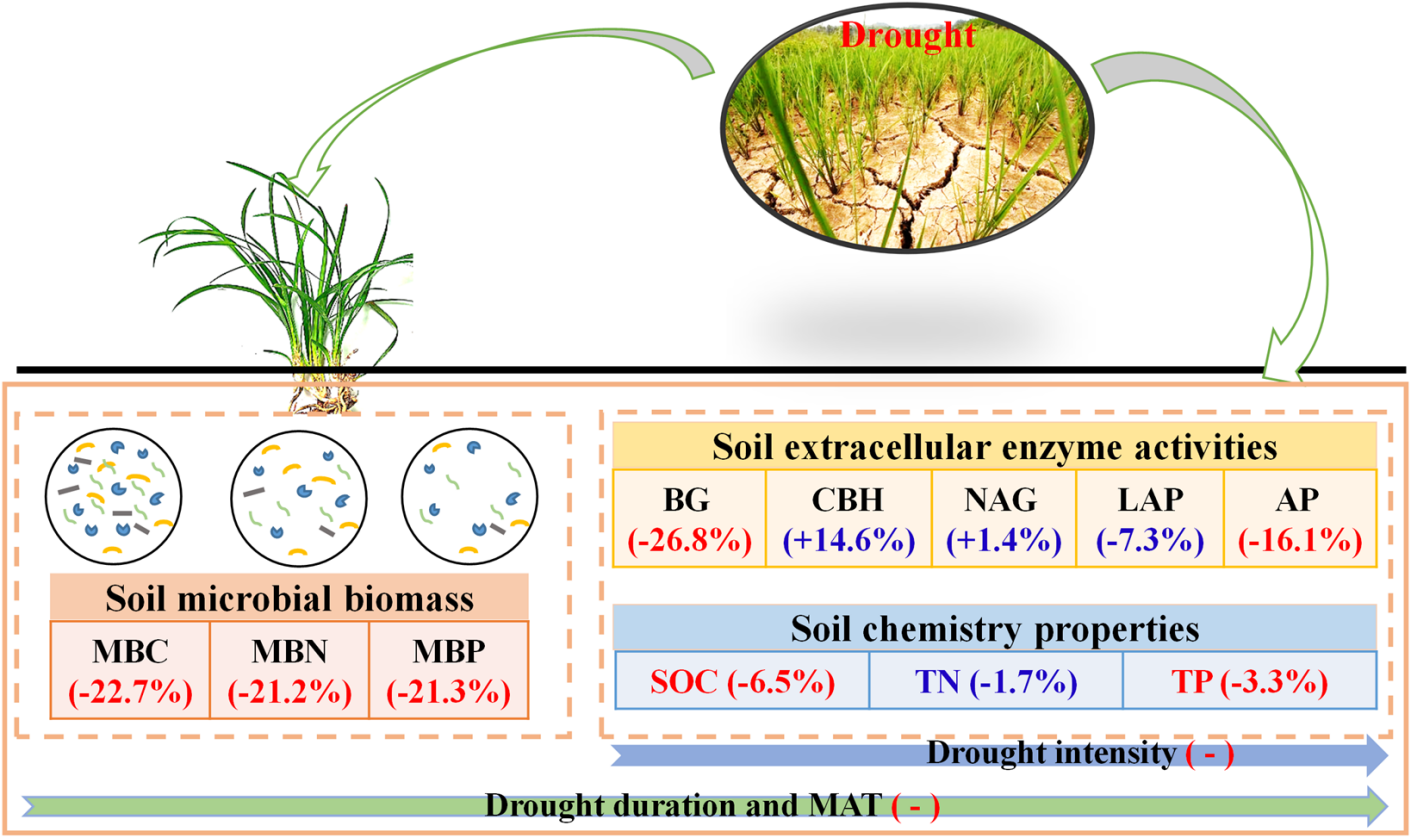
Conceptual framework showing impacts of drought on C soil microbial biomass, enzyme activity, and soil chemistry properties. “+” and “-” indicate positive and negative effects, respectively. Red and blue indicate significant and insignificant effects, respectively. Numbers in parentheses indicate percentage changes after drought. MBC, soil microbial biomass carbon. MBN, microbial biomass nitrogen. MBP, microbial biomass phosphorus. BG, β-1, 4-glucosidase. CBH, β-D-cellobiosidase. NAG, β-1, 4-N-acetylglucosaminidase. LAP, L-leucine aminopeptidase. AP, acid phosphatase. SOC, soil organic carbon. TN, total nitrogen. TP, total phosphorus.

The frequent occurrence of droughts worldwide has greatly affected the structure, function, and biodiversity of terrestrial ecosystems (Williams and de Vries, 2020; Lozano et al., 2021). However, a comprehensive understanding of the impacts of drought on microbial activity is lacking, which has limited our understanding of the multifunctional nature of ecosystems. Thus, the current study provides direct global evidence that drought has substantially reduced soil microbial biomass (MBC, 22.7%; MBN, 21.2%; MBP, 21.6%) and enzyme activity (BG, 26.8%; AP, 16.1%) (**Figure 7**). This phenomenon can be explained by the following four aspects: (1) Microorganisms have semi-permeable membranes, and the availability of soil moisture is essential for maintaining the survival and activity of microorganisms (Yan et al., 2021; Ge et al., 2022). In water-poor environments, microbial community transformation is slow, and lack of moisture may lead to microbial cracking or death (Schimel, 2018; Zhang et al., 2023). (2) Drought is generally believed to reduce plant biomass, soil litter content, and root activity, which in turn reduces the availability of food sources for microorganisms. Drought can also change the quality and amount of carbon sources available to microorganisms by reducing photosynthesis and plant growth (Malik et al., 2020b; Yan et al., 2023). Restriction of substrate concentration and availability and root exudates may be another important cause of reduced microbial activity (Ge et al., 2022; Malik and Bouskill, 2022). (3) Drought directly affects soil aeration, which in turn affects the decomposition of root exudates and organic matter and affects microbial activity and enzyme production by affecting soil physicochemical properties, and the diffusion of organic matter (Condit et al., 2013; Canarini et al., 2021; Quintana et al., 2023). (4) A decrease in soil moisture also reduces the ability of microorganisms to acquire and utilise resources (Sistla and Schimel, 2012), thus reducing their activity. Soil microorganisms are a key indicator of the ability of soils to conduct biogeochemical reactions (Battin et al., 2003; Agathokleous et al., 2020). The results of this study suggests that drought changes ecosystem structure and reduces the rate of material cycling.

### 4.2 Effect of vegetation types and continents on soil microbial biomass and enzyme activity

Owing to the complexity and heterogeneity of ecosystems, drought substantially reduced soil MBC and MBN as well as BG and AP activity in farmlands and grasslands. The results indicated that drought mainly inhibited soil microbial activity in farmlands and grasslands but did not affect microbial biomass or enzyme activity in forests to the same degree. This outcome may be related to the distribution of the sample points. Rainfall distribution varies by ecosystem, and forests are mainly distributed in areas of heavy rainfall (the MAP of all forest sample points was 1174 mm), whereas cropland and grassland are mainly distributed in areas of low rainfall (the MAP of all cropland and grassland sample points was 480 mm and 807 mm, respectively) (**Supplementary Figure 3**). Previous studies have shown that rainfall is an important factor affecting microbial activity (Ren et al., 2018; Ochoa-Hueso et al., 2020). The low MAP of cropland and grassland may have had a superimposed effect on drought, aggravating the drought stress of the soil, and thus enhancing the inhibition of plant growth and microorganism activity. In contrast, the higher MAP in forests may have alleviated the decrease in soil moisture caused by drought stress and improved the physical structure of the soil to some degree, making it more suitable for microbial growth and reproduction, thus alleviating the negative effect of drought on microbial activity. This result also indicated that soil microorganisms in croplands were more sensitive to drought responses than those in forests, as well as more susceptible to soil moisture reduction. In this study, the samples were grouped by geographical location (Asia, America, Europe, Oceania, and Africa) to explore the influence of this factor in regulating the effects of drought. Notably, we found that the effects of drought on soil microbial biomass and enzyme activity were generally similar among different locations. This contradicts the conclusion of a previous study that geographical location affected microbial nutrient restriction and thus microbial activity (Xu et al., 2022b). This discrepancy may be due to the small number of samples in America, Oceania, and Africa in this study, which reduced the statistical power of our meta-analysis results.

### 4.3 Effect of drought intensity and duration on soil microbial biomass and enzyme activity

Knowledge on the effects of high-intensity drought stress, particularly those of drought duration, on soil microbial activity is lacking. In this analysis, global-scale data on the changes in microbial activity under different drought intensities and durations were collected, and it was determined that enzyme activity decreased with increasing drought intensity, and both microbial biomass and enzyme activity decreased with increasing drought duration (**Figure 7**). We propose the following explanations for these responses to drought. First, in a water-scarce environment, microbial community activity is low; however, with the intensification of drought stress, many microorganisms die, thus reducing enzyme production (Hoover et al., 2014; Zang et al., 2020). Second, drought inhibits plant growth. Severe and long-term drought causes plants to wilt and die, thereby reducing plant biomass, which lowers the quality of available carbon sources and nutrient content for microorganisms (Smith et al., 2009; Brown et al., 2021). Third, the worsening of drought stress may lead to soil cracking, land degradation, and changes in the physical environment of the soil (air, water, aggregate structure, etc.) (Fitzpatrick et al., 2017; Wan et al., 2023), making it unsuitable for the propagation and growth of microorganisms. Additionally, studies have indicated that soil microorganisms reduce the loss of nutrients and metabolism under long-term drought stress (Manzoni et al., 2014; Brown et al., 2021), thereby reducing microbial biomass and enzyme production capacity. With the intensification of global warming, the frequency and intensity of drought events worldwide have increased (Hoover et al., 2014), which has seriously impacted the structure, function, and biodiversity of terrestrial ecosystems (Barnes et al., 2018; Qu et al., 2023). The results of this study contribute to the overall understanding of ecosystem versatility under conditions of continuous global climate change.

### 4.4 Effect of MAT on soil microbial biomass and enzyme activity

In addition to rainfall, temperature patterns also affect soil microbial activity under drought stress. In this study, microbial activity decreased with increasing MAT, and the effect of drought on microbial activity changed from positive to negative with an increase in MAT. The analysis indicated that drought had a positive effect on microbial activity in low-temperature regions, whereas microbial activity was inhibited in warmer regions. Temperature affects microbial activity by influencing the soil temperature, soil physical structure, and plant growth (Billings and Ballantyne, 2013; Abirami et al., 2021). Moderate temperature increases can promote plant growth, accelerate litter decomposition, increase soil nutrient turnover rates (Cusack et al., 2010; Joly et al., 2023), and promote the growth and reproduction of microorganisms. In contrast, excessive temperature and drought superimpose these effects and may decrease the availability of soil water and reduce the turnover rate of soil nutrients (Xu et al., 2022b; Qu et al., 2023), thereby strongly inhibiting microbial activity. Soil microorganisms play an important role in material cycling in terrestrial ecosystems (Agathokleous et al., 2020). The results of this study highlight the impact of drought on soil microbial biomass and enzyme activity and the resulting effects on nutrient cycling processes in terrestrial ecosystems.

## 5 Conclusions

Our integrated analysis provided direct evidence that drought has significantly inhibited soil microbial activity globally. In particular, drought had a stronger inhibitory effect on soil microbial biomass and soil carbon- and phosphorus-acquiring enzyme activity and a weaker effect on soil nitrogen-acquiring enzyme activity. Furthermore, drought had a greater effect on microbial biomass than on soil enzyme activity. Additionally, our results revealed a negative correlation between microbial activity and drought intensity, drought duration, and MAT. Our results contribute to the overall understanding of the structure and function of terrestrial ecosystems under global climate change.

## Data availability statement

The original contributions presented in the study are included in the article/**Supplementary Material**, further inquiries can be directed to the corresponding author/s.

## Author contributions

QQ, RL, and HX conceived and designed this meta-analysis. QQ, QG, and ZW analyzed the data. QQ drafted the original manuscript. RL and HX helped analyze the data and rewrote part of the manuscript. All authors contributed to the article and approved the submitted version.
