# Corrigendum: Impact of drought on soil microbial biomass and extracellular enzyme activity

**DOI:** 10.3389/fpls.2024.1393574

**Published:** 2024-03-21

**Authors:** Qing Qu, Zhen Wang, Quan Gan, Rentao Liu, Hongwei Xu

**Affiliations:** ^1^Breeding Base for State Key Laboratory of Land Degradation and Ecological Restoration in Northwestern China, Key Laboratory of Restoration and Reconstruction of Degraded Ecosystems in Northwestern China of Ministry of Education, Ningxia University, Yinchuan, China; ^2^Institute of Soil and Water Conservation, Chinese Academy of Sciences and Ministry of Water Resources, Yangling, China; ^3^College of Forestry, Sichuan Agricultural University, Chengdu, China

**Keywords:** biogeochemical cycles, climate change, ecosystem function, ecosystem structure, soil microbial activity, soil microbial community

**Text Correction**

In the published article, there was an error. In the published article, there was a mistake in the formula. The formula was displayed as “SD =√SE”. The correct statement is “ $\text{SD}=\text{SE}\sqrt{\text{N}}$ ”.

A correction has been made to 2 Materials and methods, 2.1 Data collection. This sentence previously stated:

“If no standard deviation was reported, it was calculated using the standard error (SE) as follows: SD =√SE (Fu et al., 2015).”

The corrected sentence appears below:

“If no standard deviation was reported, it was calculated using the standard error (SE) as follows: $\text{SD}=\text{SE}\sqrt{\text{N}}$ (Fu et al., 2015).”

The authors apologize for this error and state that this does not change the scientific conclusions of the article in any way. The original article has been updated.

