# Supplementary material for: Impact of drought on soil microbial biomass and extracellular enzyme activity
Source: Front Plant Sci. 2023 Aug 25;14:1221288. doi: 10.3389/fpls.2023.1221288 (PMC10491016; doi:10.3389/fpls.2023.1221288)
Supplement: Supplementary file 1 [file DataSheet_1.docx]

Supplementary Table 1 The search terms used in this study.

| Keywords 1 |  | Keywords 2 |
| --- | --- | --- |
| Drought, precipitation exclusion, decreased precipitation, decreased rainfall, throughfall reduction, water stress | **AND** | soil carbon, soil nitrogen, soil phosphorus, soil nutrients, soil properties, soil microbial activity, soil microbial biomass, soil enzyme activity |

Supplementary Table 2 12 variables included in this study.

|  | Variables |
| --- | --- |
| Soil chemical | pH, soil organic carbon (SOC), soil total nitrogen (TN), soil total phosphorus (TP) |
| Soil microbial | soil microbial biomass carbon (MBC), soil microbial biomass nitrogen (MBN), soil microbial biomass phosphorus (MBP) |
| Enzyme | β-1, 4-glucosidase (BG), β-D-cellobiosidase (CBH), β-1, 4-N-acetylglucosaminidase (NAG), L-leucine aminopeptidase (LAP), acid phosphatase (AP) |

Supplementary Table 3 Effect test summary of ecosystem type on each response variable of soil microbial biomass, enzyme, and soil under the random effects model.

|  | MBC | MBN | MBP | BG | CBH | NAG | AP |
| --- | --- | --- | --- | --- | --- | --- | --- |
| QM | 28.59 | 68.44 | 49.40 | 38.20 | 8.04 | 16.80 | 24.56 |
| p | <0.001 | <0.001 | <0.001 | <0.001 | 0.018 | <0.001 | <0.001 |
|  | SOC | TN | TP | pH |  |  |  |
| QM | 72.75 | 12.95 | 41.28 | 19.25 |  |  |  |
| p | <0.001 | 0.012 | <0.001 | <0.001 |  |  |  |

Notes: Q_M_, heterogeneity in group cumulative effect sizes. MBC, soil microbial biomass carbon. MBN, microbial biomass nitrogen. MBP, microbial biomass phosphorus. BG, β-1, 4-glucosidase. CBH, β-D-cellobiosidase. NAG, β-1, 4-N-acetylglucosaminidase. LAP, L-leucine aminopeptidase. AP, acid phosphatase. SOC, soil organic carbon. TN, total nitrogen. TP, total phosphorus.

Supplementary Table 4 Effect test summary of continent on each response variable of soil microbial biomass, enzyme, and soil under the random effects model.

|  | MBC | MBN | MBP | BG | CBH | NAG | LAP | AP |
| --- | --- | --- | --- | --- | --- | --- | --- | --- |
| QM | 3.35 | 36.63 | 9.72 | 2.85 | 11.27 | 1.13 | 32.40 | 24.63 |
| p | 0.356 | <0.001 | 0.210 | 0.555 | 0.100 | 0.180 | <0.001 | <0.001 |
|  | SOC | TN | TP | pH |  |  |  |  |
| QM | 1.64 | 7.60 | 4.42 | 13.36 |  |  |  |  |
| p | 0.590 | 0.179 | 0.352 | 0.400 |  |  |  |  |

Notes: Q_M_, heterogeneity in group cumulative effect sizes. MBC, soil microbial biomass carbon. MBN, microbial biomass nitrogen. MBP, microbial biomass phosphorus. BG, β-1, 4-glucosidase. CBH, β-D-cellobiosidase. NAG, β-1, 4-N-acetylglucosaminidase. LAP, L-leucine aminopeptidase. AP, acid phosphatase. SOC, soil organic carbon. TN, total nitrogen. TP, total phosphorus.


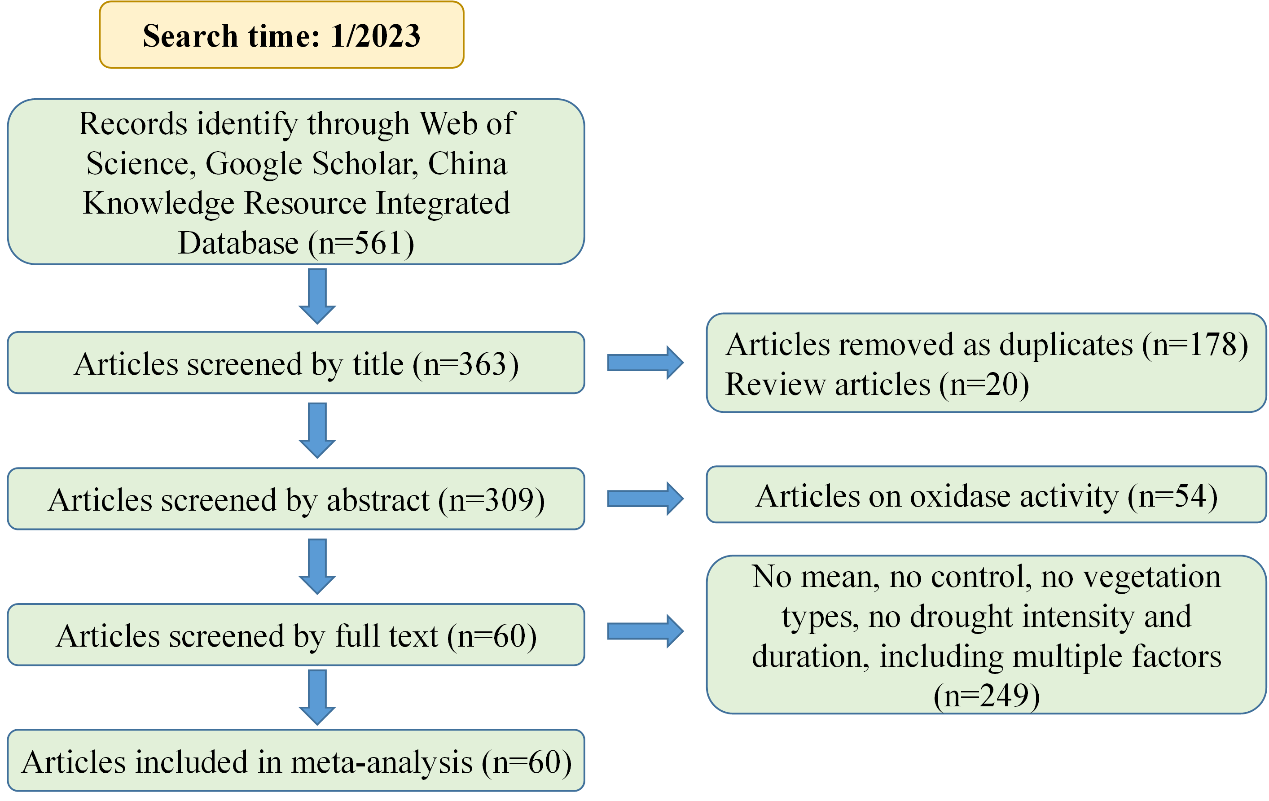


Supplementary Figure 1. Flow diagram showing steps in study filtering strategy.


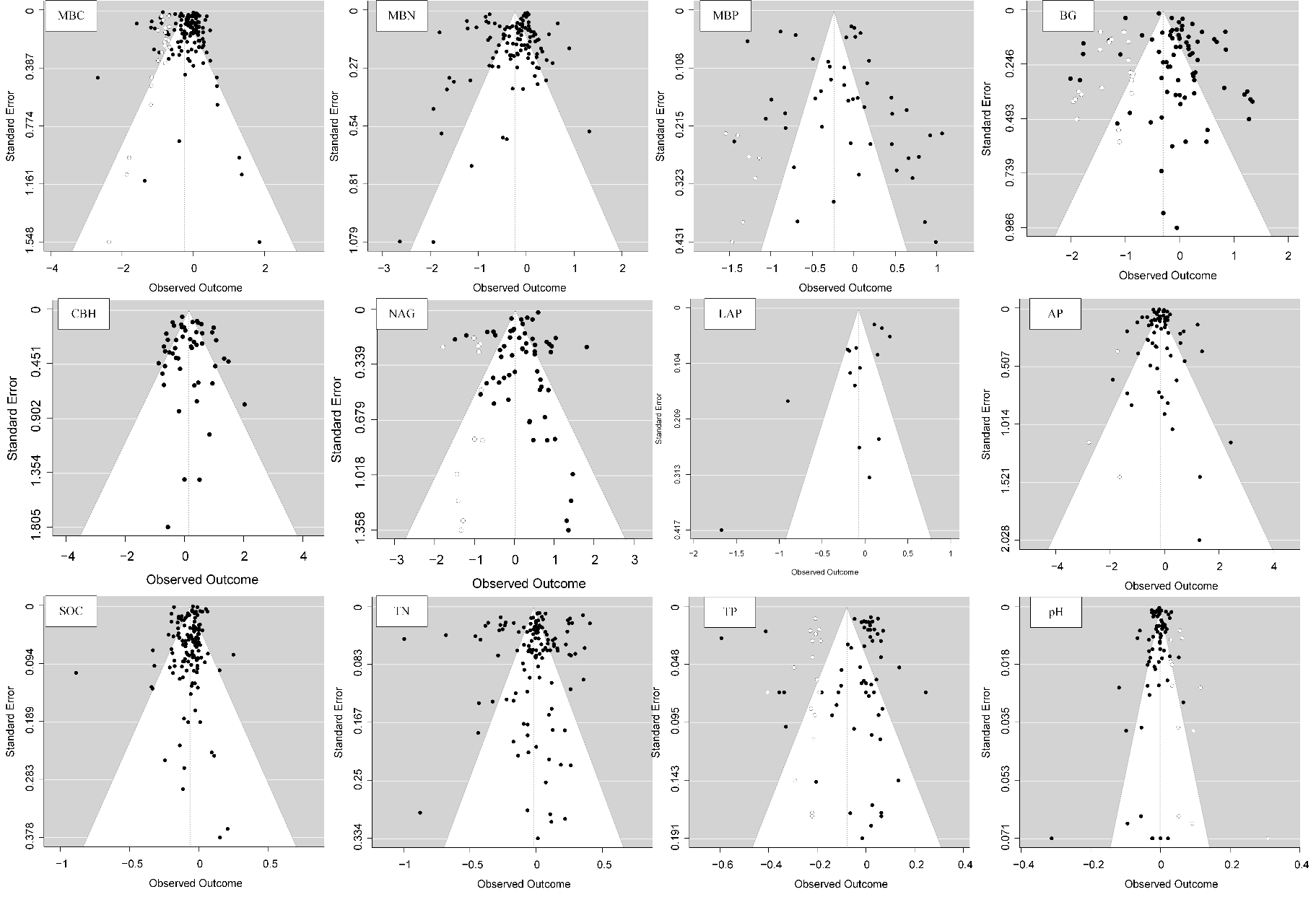


Supplementary Figure 2. The funnel plot of the response ratios of soil microbial biomass, enzyme activity, and soil chemistry properties under drought. Notes: MBC, soil microbial biomass carbon. MBN, microbial biomass nitrogen. MBP, microbial biomass phosphorus. BG, β-1, 4-glucosidase. CBH, β-D-cellobiosidase. NAG, β-1, 4-N-acetylglucosaminidase. LAP, L-leucine aminopeptidase. AP, acid phosphatase. SOC, soil organic carbon. TN, total nitrogen. TP, total phosphorus.





Supplementary Figure 3. Changes in annual average temperature (MAT) and mean annual precipitation (MAP).
